# Progression from laparoscopic-assisted to totally laparoscopic distal gastrectomy: comparison of circular stapler (i-DST) and linear stapler (BBT) for intracorporeal anastomosis

**DOI:** 10.1007/s00464-012-2433-y

**Published:** 2012-06-26

**Authors:** Tetsuo Ikeda, Hiroyuki Kawano, Yuichi Hisamatsu, Koji Ando, Hiroshi Saeki, Eiji Oki, Takefumi Ohga, Yoshihiro Kakeji, Shunichi Tsujitani, Shunji Kohnoe, Yoshihiko Maehara

**Affiliations:** Department of Surgery and Science, Graduate School of Medical Sciences, Kyushu University, 3-1-1 Maidashi, Higashi-ku, Fukuoka 812-8582 Japan

**Keywords:** Laparoscopic distal gastrectomy, Totally laparoscopic gastrectomy, Intracorporeal anastomosis, Intracorporeal DST, Book-binding technique

## Abstract

**Background:**

Billroth I (B-I) gastroduodenostomy is an anastomotic procedure that is widely performed after gastric resection for distal gastric cancer. A circular stapler often is used for B-I gastroduodenostomy in open and laparoscopic-assisted distal gastrectomy. Recently, totally laparoscopic distal gastrectomy (TLDG) has been considered less invasive than laparoscopic-assisted gastrectomy, and many institutions performing laparoscopic-assisted distal gastrectomy are trying to progress to TLDG without markedly changing the anastomosis method. The purpose of this report is to introduce the technical details of new methods of intracorporeal gastroduodenostomy using either a circular or linear stapler and to evaluate their technical feasibility and safety.

**Methods:**

Seventeen patients who underwent TLDG with the intracorporeal double-stapling technique using a circular stapler (*n* = 7) or the book-binding technique (BBT) using a linear stapler (*n* = 10) between February 2010 and April 2011 were enrolled in the study. Clinicopathological data, surgical data, and postoperative outcomes were analyzed.

**Results:**

There were no intraoperative complications or conversions to open surgery in any of the 17 patients. The usual postoperative complications following gastroduodenostomy, such as anastomotic leakage and stenosis, were not observed. Anastomosis took significantly longer to complete with DST (64 ± 24 min) than with BBT (34 ± 7 min), but more stapler cartridges were needed with BBT than with DST.

**Conclusions:**

TLDG using a circular or linear stapler is feasible and safe to perform. DST will enable institutions performing laparoscopic-assisted distal gastrectomy with circular staplers to progress to TLDG without problems, and this progression may be more economical because fewer stapler cartridges are used during surgery. However, if an institution has already been performing δ anastomosis in TLDG but has been experiencing certain issues with δ anastomosis, converting from δ anastomosis to BBT should be beneficial.

**Electronic supplementary material:**

The online version of this article (doi:10.1007/s00464-012-2433-y) contains supplementary material, which is available to authorized users.

Billroth-I (B-I) gastroduodenostomy has long been preferred for reconstruction after distal gastrectomy, because it affords the physiological advantage of allowing food to pass through the duodenum and the endoscopic observation and treatment of the duodenum and biliary tract. Therefore, B-I also has been widely performed after laparoscopic-assisted distal gastrectomy (LADG) [1–8]. LADG is the most commonly performed procedure for the treatment of early gastric cancer and involves laparoscopic mobilization of the stomach and systematic lymph node dissection. However, subsequent reconstruction is performed with direct visualization through a small incision made in the epigastrium. Recently, totally laparoscopic distal gastrectomy (TLDG) has been considered less invasive than LADG [9–15], and many institutions performing LADG are trying to progress to TLDG without markedly changing the anastomosis method.

In more than 100 cases of LADG, we have performed intracorporeal B-I stapled anastomosis using a hand-access device according to a method similar to that reported by Joo et al. [16]. In this method, under direct visualization through a small incision made in the epigastrium, a pursestring suture is applied by using a handsewing technique and the anvil of the circular stapler is inserted into the duodenal stump. Once the stomach has been pulled out to the extraperitoneal space, a hole is made on the oral side of the cancer lesion, and a circular stapler is inserted through this hole. The stomach, along with the inserted circular stapler, is then reinserted into the peritoneal cavity, and a laparoscopic hand access device is closed over the stomach and the shaft of the circular stapler. Pneumoperitoneum is reestablished, and the posterior gastric wall located on the oral side of the cancer lesion is then punctured with the central rod of the circular stapler. The anvil head previously inserted into the duodenal stump is attached to the central rod and fired under laparoscopy. Finally, the stomach, including the cancer lesion is transected on the anal side of the anastomotic site.

In this method, anastomosis is performed laparoscopically; however, it is difficult to apply a pursestring suture at the duodenal stump in obese patients and in those with a thickened abdominal wall or distorted perigastric anatomy. Because the stomach is transected after the anastomosis, confirming proper resection of the lesion is not always possible. Furthermore, because the anastomosis is performed between the posterior gastric wall and the duodenal stump, excessive tension may occur, which often requires that the anterior wall of the anastomosis be reinforced by hand sewing. For these reasons, we decided to progress to TLDG in B-I gastroduodenostomy and determine which of these two techniques is optimal.

One of the two techniques examined in this study is similar to the conventional method used at our institution, but it can overcome the problems associated with the conventional method. The other technique is completely different from the conventional method, but addresses issues associated with δ anastomosis, which was introduced by Kanaya et al. [17]. The δ anastomosis has been widely used as the only anastomotic procedure in totally laparoscopic B-I gastroduodenostomy, because it can be completed in a short time with wide anastomosis. On the other hand, completion of δ anastomosis requires at least six cartridges. Moreover, it is necessary to perform additional dissection of the duodenum, which tends to become ischemic. These have been pointed out as unacceptable issues by some laparoscopic surgeons.

## Materials and methods

### Patients

Seventeen patients who underwent TLDG with the intracorporeal double-stapling technique (i-DST; *n* = 7) or the book-binding technique (BBT; *n* = 10) between February 2010 and April 2011 were enrolled in this study. Candidates for TLDG were patients preoperatively staged as T1N0 or T1N1 according to endoscopic ultrasonography and abdominal computed tomography results. Roux-en-Y reconstruction was performed in the following cases: a lesion was present around the pyloric ring, there was an increased risk of recurrence in the pyloric ring surroundings, the remnant stomach was smaller than 20–25 % of the entire stomach, or the tumor had infiltrated the duodenum. Clinicopathological data, surgical data, and postoperative outcomes were analyzed for all patients.

### Surgical technique

**Fig. 1:**
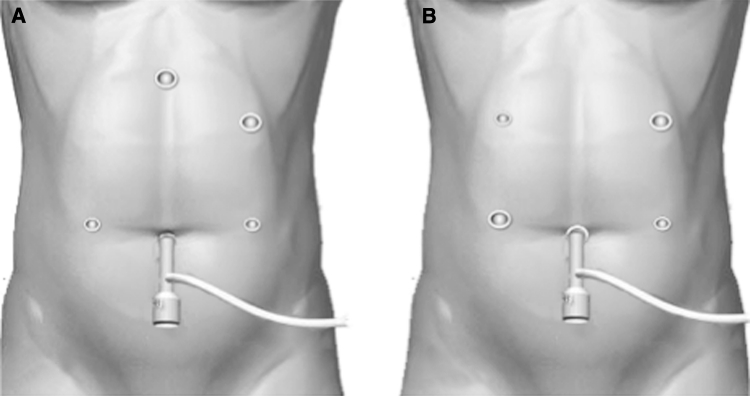
Illustration of trocar placement for totally laparoscopic Billroth-I gastrectomy with **A** i-DST and **B** BBT. A 12-mm trocar was inserted through an umbilical incision, and four other trocars (two 12-mm trocars and two 5-mm trocars) were placed under laparoscopic guidance

**Fig. 2:**
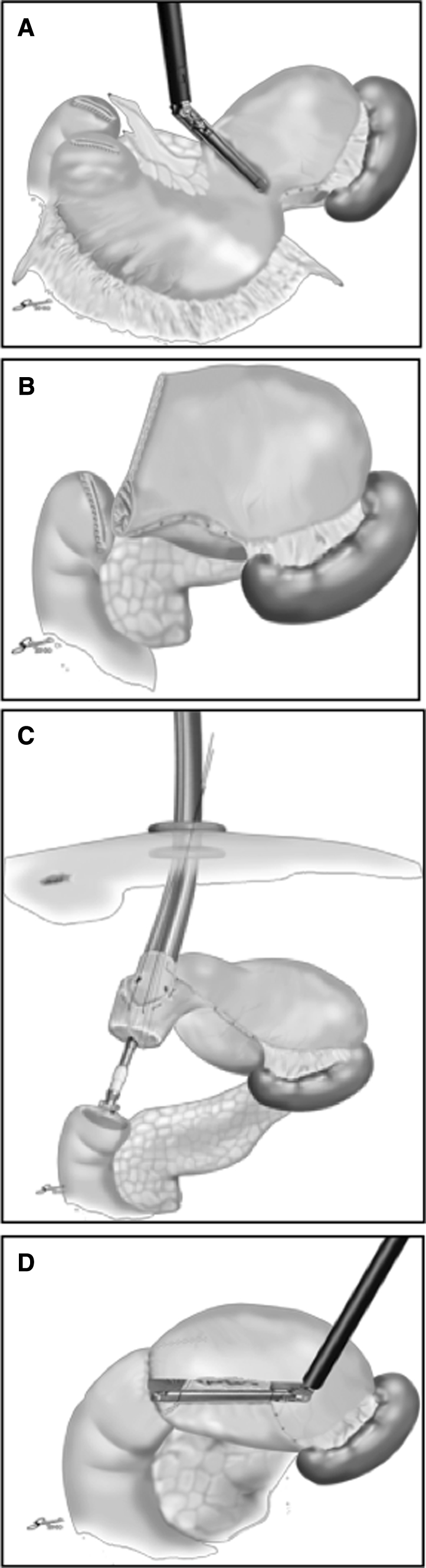
Illustration of the i-DST. **A** 60-mm linear stapler was introduced through the epigastric midline port, and resection of the stomach was performed only once. **B** The distal side of the stomach was completely transected using bipolar scissors. **C** The circular stapler was introduced into the remnant stomach, attached to the previously inserted duodenal anvil head, and fired. **D** The unclosed part of the remnant stomach was closed with the linear stapler

**Fig. 3:**
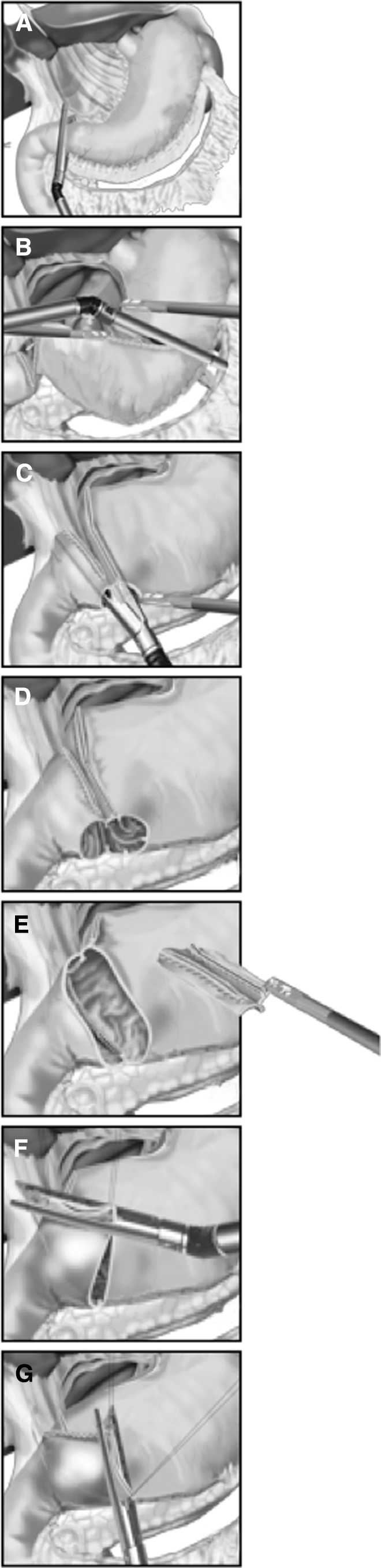
Illustration of the BBT. **A** The duodenal bulb was transected just below the pyloric ring from the greater curvature side toward the lesser curvature side. **B** The stomach was transected from the lesser curvature side toward the greater curvature side. **C** 45-mm endoscopic linear stapler was inserted through the left lower port, and a jaw was inserted into each of the created holes. **D** After the first stapling, there were three staple lines including those from the transection of the stomach and duodenum, which ran parallel to the anterior wall. **E** All of the transection lines on the duodenum, anterior side of the anastomosis line between the duodenum and stomach, and approximately one-third of the transection line on the stomach were dissected, and holes were made in the anterior wall. **F** The anterior hole was closed from the center to the lesser curvature. **G** The remaining hole from the greater curvature to the center was then closed

Under general anesthesia, the patient was placed in the supine position with the legs apart (Figs. 1, 2, 3). The surgeon was positioned on the right side of the patient with the camera operator between the patient’s legs, and the first assistant was positioned on the left side of the patient. During the operation, the surgeon and the first assistant switched their positions as needed. A 12-mm trocar was inserted through an umbilical incision using an open technique, and pneumoperitoneum was established. Four other trocars (two 12-mm and two 5-mm) were placed under laparoscopic guidance (Fig. 1). After mobilization of the gastroduodenum, the duodenal bulb was transected just below the pyloric ring from the greater curvature side toward the lesser curvature side using an endoscopic linear stapler (Endo GIA; Covidien Ltd, Norwalk, CT; Fig. 3A).

Sufficient lymphadenectomy was performed, and the stomach was transected from the lesser curvature side toward the greater curvature side using the endoscopic linear stapler (Fig. 3B). In the i-DST group, a 60-mm linear stapler with a green cartridge (staple leg length, 4.5 mm) was introduced through the epigastric midline port. Usually, resection of the stomach with this stapler is performed only once (Fig. 2A), because three-quarters of the stomach can be transected during the first stapling and the undivided part of the greater curvature side of the stomach is useful for insertion of the circular stapler for gastroduodenostomy. The distal side of the undivided stomach was then closed with an endoscopic intestinal clamp, and transection was completed using bipolar scissors (Fig. 2B). In the BBT group, the stapler was introduced through the right lower port and the stomach was transected with two 60-mm linear staplers. In the i-DST group, the epigastric midline incision was extended transversally by approximately 3.5 cm. In the BBT group, the umbilical incision was extended longitudinally by approximately 3.5 cm. Thereafter, the divided stomach and surrounding tissue were removed using a large plastic bag. The pneumoperitoneum device was then attached to the extended epigastric midline incision or umbilical incision.

### Intracorporeal gastroduodenostomy for i-DST

After the pneumoperitoneum was reestablished, the stapler line on the duodenal stump was removed, a pursestring suture was placed with intracorporeal handsewing, and a 31-mm anvil for the circular stapler was typically inserted into the duodenal stump (Fig. 2). A pursestring suture also was placed at the unclosed part of the greater curvature of the remnant stomach. Through the attached pneumoperitoneum device, the circular stapler was introduced into the unclosed part of the greater curvature of the remnant stomach. Because the remnant stomach must be twisted at approximately 90°, the relationship between the greater curvature and the lesser curvature changed from right and left to top and bottom. A traction suture was placed at the opening of the anvil using 2-0 silk, and just dorsal to the transection staple line, the central rod of the circular stapler punctured through the gastric wall. The previously inserted duodenal anvil head was then attached to the central rod, and the stapler was fired (Fig. 2C). The unclosed part of the remnant stomach was closed with the linear stapler to conclude the intracorporeal B-I anastomosis (Fig. 2D).

### Intracorporeal gastroduodenostomy for BBT

**Fig. 4:**
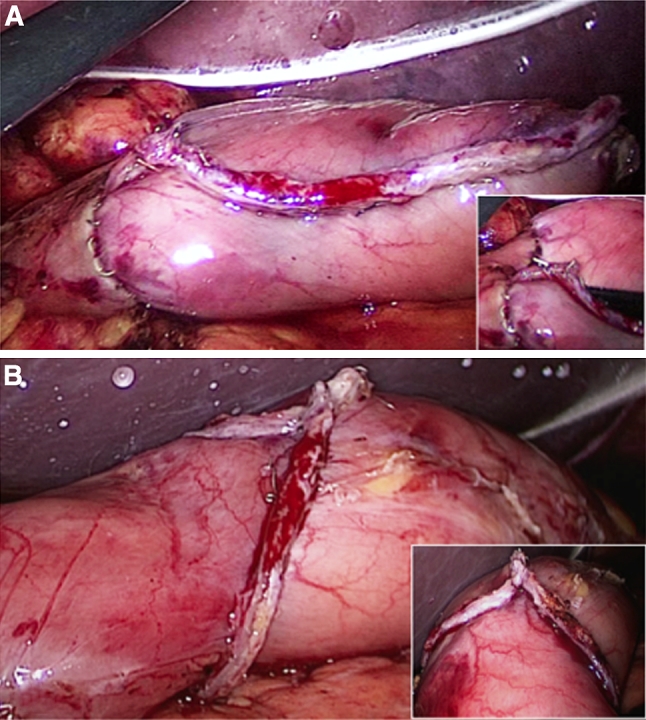
**A** Laparoscopic view of the completed anastomosis with i-DST. **B** Laparoscopic view of the completed anastomosis with BBT

Small entry holes were made on the greater curvature side of the stomach and the duodenal stump (Fig. 3). The entry hole on the stomach side was made on the transection staple line, whereas the entry hole on the duodenal stump was made on the greater curvature side. A 45-mm endoscopic linear stapler was inserted through the left lower port, and a jaw was inserted into each of the created holes (Fig. 3C). The stapler was closed and fired just behind the staple lines of both stumps. Because the duodenal stump was often shorter than 45 mm, the intraduodenal jaw tip was advanced to just behind the duodenal lesser curvature. Thus, the remnant stomach and duodenum were attached just behind the staple lines of both stumps to form the base of the triangular anastomosis. At this point, there were three staple lines, including those from the transection of the stomach and duodenum (one introverted line in the middle and two extroverted lines on the sides), which ran parallel to the anterior wall (Fig. 3D). Using bipolar scissors, all transection lines on the duodenum, anterior side of the anastomosis line between the duodenum and stomach, and approximately one-third of the transection line on the stomach were dissected, and holes were made in the anterior wall (Fig. 3E). Next, these holes on the anterior wall, including the entry hole of the linear stapler, were anastomosed together by using two applications of the linear stapler so that they formed the tip of a triangle. Specifically, using three stay sutures at the center and at both ends as support, the anastomosis was completed by first closing the center to the lesser curvature (Fig. 3F) and then closing from the greater curvature to the center (Fig. 3G). The integrity of the closure was tested while immersed in normal saline by infusing air into the pouch lumen via a nasogastric tube and looking for escaping bubbles. The completed anastomosis of each technique is shown in Fig. 4.

## Results

A total of 17 TLDG with B-I gastrectomy (10 BBTs and 7 i-DSTs) were performed, and no conversion to open surgery was recorded. The clinical backgrounds of the patients are summarized in Table 1. There were no significant differences in age, sex, body mass index, or presence of comorbidities between the two groups. The clinical and pathological cancer findings are listed in Table 1. Details of the operative status are given in Table 2. The mean operating time did not significantly differ between the two procedures (288 ± 45 min for DST, 255 ± 13 min for BBT), but the mean time required for anastomosis with i-DST (64 ± 24 min) differed significantly from that with BBT (34 ± 7 min). BBT anastomosis required more surgical end-stapler reloads than did i-DST anastomosis (2.2 ± 0.6 for DST, 3.0 ± 0 for BBT; *P* < 0.05). Estimated blood loss and range of the number of harvested lymph nodes did not differ between the two groups. Details of postoperative recovery for the two groups are given in Table 2. The time to first flatus and postoperative hospital stay did not differ significantly between the groups. The usual postoperative complications following gastroduodenostomy, such as anastomosis leakage and stenosis, were not observed in either group.

**Table 1:** Clinical characteristics of the 16 patients

Factors	i-DST (circular stapler) (*n* = 7)	BBT (linear stapler) (*n* = 9)
Age [range (mean)]	45–76 (59.7)	35–84 (59.3)
Male:female ratio	5:2	6:3
BMI (kg/m^2^)	25.4 ± 3.1	23.1 ± 3.4
Tumor localization [*n* (%)]		
MU	1	1
M	6	5
ML	0	0
L	0	3

*i-DST* intracorporeal double stapling technique, *BBT* book binding technique

**Table 2:** Surgical results and postoperative course

Factors	i-DST (circular stapler) (*n* = 7)	BBT (linear stapler) (*n* = 9)	*p* value
Operating time (min)	288 ± 45	255 ± 13	NS
Anastomotic time (min)	64 ± 24	34 ± 7	0.043
Estimated blood loss (g)	58 ± 43	50 ± 66	NS
Transfusion [*n* (%)]	0 (0)	0 (0)	
Open conversion [*n* (%)]	0 (0)	0 (0)	
Liquid diet (day)	4.8 ± 0.8	5.1 ± 0.4	NS
First flatus (day)	1.3 ± 0.9	1.8 ± 0.4	NS
Complication [*n* (%)]	0 (0)	0 (0)	
Mortality [*n* (%)]	0 (0)	0 (0)	
Postoperative hospital stay (day)	11.3 ± 0.8	14.2 ± 2.3	NS
No. of harvested lymph nodes	26 ± 11.3	29.3 ± 14.1	NS
Stage Ia:Ib	6:1	7:2	

*i-DST* intracorporeal double stapling technique, *BBT* book binding technique, *NS* not significant

## Discussion

In 116 patients between June 2004 and December 2009, LADG was performed with intracorporeal B-I stapled anastomosis using a circular stapler. None of the 116 patients developed obvious anastomotic leakage (one patient developed a subphrenic abscess), and only one patient developed postoperative stasis that resulted in the cessation of oral feeding. At the time of moving to perform TLDG, we recalled an anastomotic procedure similar to LADG in which the anastomosis was created using a circular stapler. To the best of our knowledge, there are only two case reports concerning intracorporeal anastomosis using a circular stapler in TLDG, and no studies with a large number of cases have been reported [18, 19].

We have performed TLDG with DST in seven cases thus far. In our attempts, we realized that after removing two-thirds to three-quarters of the stomach, intracorporeal insertion of the circular stapler into the remnant stomach was not easy, even from the epigastric region. Some ingenuity is required when inserting the circular stapler and attaching it to the anvil head in the duodenal stump, and pursestring sutures are then applied over the entry hole for the circular stapler on the remnant stomach. Furthermore, stay sutures must be placed on the contralateral side of the planned anastomosis site so that the central rod of the circular stapler is punched correctly through the stomach just dorsal to the transection line. By doing so, a double-stapling anastomosis just dorsal to and near the center of the transection line can be successfully completed. In this method, because the anastomosis is completed intracorporeally, it is not necessary to extend the incision to a size larger than that necessary to insert the 31-mm circular stapler and extract the specimen. The size of the incision was small enough to open by splitting the rectus abdominis muscle without dissection. This is in contrast to all of the 116 LADG cases of intracorporeal B-I stapled anastomosis in which a hand-access device [16] was needed to dissect the rectus muscle and among which 18 of the 116 patients underwent extension of the incision beyond 7 cm because the patients were obese or had a thickened abdominal wall. In these patients, it was necessary to extend the incision to the right, thus transecting the rectus abdominis with a higher chance of damaging local nerves. Furthermore, in LADG using a circular stapler, examination for bleeding at the anastomosis site was difficult, and we experienced three cases of postanastomotic bleeding requiring blood transfusion. In TLDG with DST, the anastomosis sites were clearly visible from the opening for the circular stapler, and it was therefore possible to stop bleeding during surgery.

The δ anastomosis technique, a B-I gastrectomy procedure performed in TLDG, was first introduced by Kanaya et al. [17]. This technique is relatively simple, because it involves a wider lumen anastomosis and has a lower incidence of complications. Despite the fact that the technique has been widely used in Japan and South Korea, it is associated with certain problems that make its incorporation challenging. One of the problems associated with the technique is that because the duodenum needs to be transected from the posterior wall toward the anterior wall, the anastomotic method must be predetermined before transecting the duodenum. Another problem is that because the duodenum must be twisted for anastomosis, not only must the greater curvature side of the duodenum be detached, but the dorsal and the lesser curvature sides must be completely detached as well. As the name of the procedure implies, the anastomosed site forms a δ shape, and the blood flow through the duodenal stump forming the apex must be decreased. In addition, because both the stomach and duodenum are twisted and their posterior walls are anastomosed, more tension is generated in the anastomosis compared with end-to-end anastomosis.

For intracorporeal B-I anastomosis in TLDG, the triangulating stapling technique was reported by Tanimura et al. [20]. In this method, the duodenum is transected in any direction, and by forming a triangle, the anastomosis lumen is made wide with no ischemic areas. However, the first introverted anastomosis, which forms the base of the triangle, is cumbersome once all of the staple lines on the stomach and duodenum have been cut off. There are some differences between the stomach and duodenum in terms of lumen size, wall thickness, and wall extensibility. For these reasons, in the first anastomoses, it can be difficult to connect accurately the posterior walls and the following anterior wall anastomoses.

To address these problems, BBT anastomosis using linear staplers focuses on the following points. First, instead of completely opening the stomach and duodenal stumps, small openings are made just wide enough to insert one of the jaws of the linear stapler. The first anastomosis is then performed, which forms the base of the triangular anastomosis, by anastomosing the immediate posterior wall of each stump to create an anastomosis similar to that formed by end-to-end anastomosis. At this point, the four layers of the gastroduodenal wall look like a bound book; therefore, we named this method the book-binding technique.

After forming the base of the triangle, to prevent the formation of ischemic areas, a large opening was created on the anterior wall by transecting the entire duodenal stump and one-third of the gastric stump (because the gastric wall stretches easily) together with the anterior wall of the first anastomosis line. A linear stapler was then fired twice to close the large opening as if forming an isosceles triangle with the tip pointing toward the stomach. A total of 17 TGL with B-I gastrectomy (10 BBTs, 7 i-DSTs) were performed, and no conversion to open surgery was required. The anastomotic technique did not lead to conversion to other techniques, and surgical incisions remained just wide enough to insert anastomotic instruments and remove the resected specimen. Moreover, no anastomotic leakage or stenosis was observed with either technique.

There are some differences between i-DST and BBT. More time is required for anastomosis in i-DST than in BBT. It takes time to complete DST in TLDG, because it is necessary to apply stay sutures to the remnant stomach not only to insert the anvil head into the duodenal stump, but also to puncture the central rod through the target site on the remnant stomach by lifting the stomach in the intraperitoneal cavity. The number of stapler cartridges used in the two anastomosis techniques also differs. While one circular stapler and three linear stapler cartridges are required in i-DST, it is necessary to use at least six linear stapler cartridges in BBT, demonstrating that i-DST is slightly more economical than BBT.

**Fig. 5:**
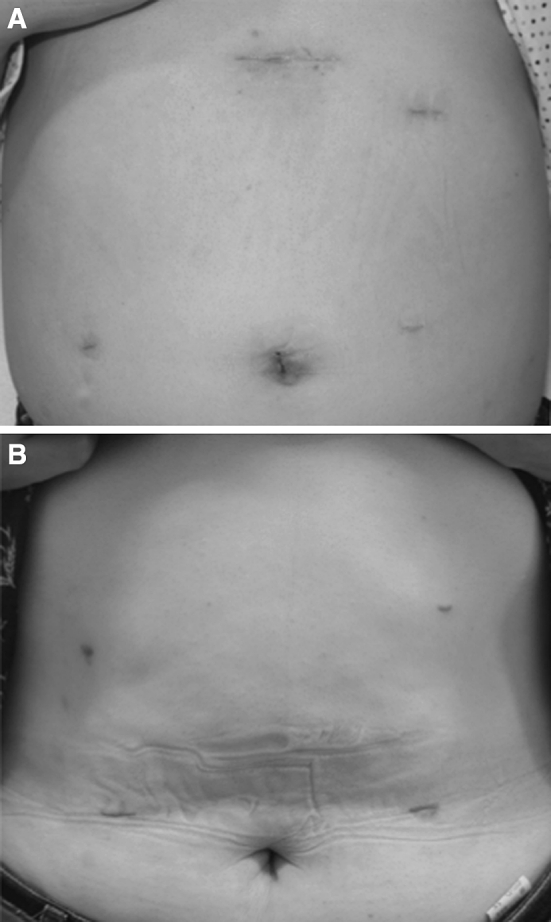
**A** Postoperative wounds of the patient who underwent TLDG with i-DST. **B** Postoperative wounds of the patient who underwent TLDG with BBT

Another difference between the i-DST and BBT techniques concerns the purpose and site for extending the incision (Fig. 5). With BBT (Fig. 5A), an incision can be made anywhere, because the purpose of extending the incision is to remove the resected specimen; therefore, an umbilical incision is generally used because it is scarcely noticeable. With DST (Fig. 5B), the incision needs to be extended not only to remove the resected specimen, but also to insert the circular stapler in the proper orientation. For the latter purpose, the incision needs to be located in the upper abdominal region. As explained earlier, we designed the B-I techniques so that i-DST using the circular stapler can overcome the problems associated with LADG. In addition, BBT using the linear stapler can resolve the issues associated with δ anastomosis, which is widely used as the only B-I anastomosis procedure in TLDG at present. Although it is not yet clear which of the two techniques is superior because of the small number of surgeries that we have performed so far, BBT, which does not require intracorporeal handsewn pursestring sutures and does not require expansion of the incision in the upper abdomen, is considered to be a more appropriate method during the introductory period of TLDG. Moreover, the time needed for anastomosis likely can be shortened with experience, especially in BBT compared with i-DST.

In conclusion, TLDG with a circular or linear stapler is feasible and safe. For institutions that have been performing LADG using circular staplers, it might be easier to progress to TLDG with i-DST. However, for institutions that have already been performing the δ anastomosis technique in TLDG but have experienced certain issues with it, it may be beneficial to convert from δ anastomosis to BBT.

## Electronic supplementary material

Below is the link to the electronic supplementary material.

**Video 1** The movie file named i-DST shows a short video summarizing the anastomosis with i-DST (WMV 15635 kb)

**Video 2** The movie file named BBT shows a short video summarizing the anastomosis with BBT (WMV 15129 kb)
